# Vancomycin Wrap for Anterior Cruciate Ligament Surgery: Molecular Insights

**DOI:** 10.1177/0363546520981570

**Published:** 2021-01-06

**Authors:** Caroline M. Atherton, Simon J. Spencer, Katy McCall, Emma Garcia-Melchor, William J. Leach, Michael Mullen, Brian P. Rooney, Colin Walker, Iain B. McInnes, Neal L. Millar, Moeed Akbar

**Affiliations:** * Institute of Infection, Immunity and Inflammation, College of Medicine, Veterinary and Life Sciences University of Glasgow, Glasgow, UK; † Department of Orthopaedic Surgery, Queen Elizabeth University Hospital Glasgow, Glasgow, UK; Investigation performed at the University of Glasgow, Glasgow, UK

**Keywords:** ACL, anterior cruciate ligament, inflammation, vancomycin, vancomycin wrap

## Abstract

**Background:**

The use of the vancomycin wrap to pretreat the hamstring graft in anterior cruciate ligament reconstruction (ACLR) has grown in popularity since it was first described in 2012 and has significantly reduced rates of postoperative infection. However, it remains unknown if this antibiotic treatment affects the molecular composition of the graft.

**Purpose:**

To establish whether treatment with vancomycin at 5 mg/mL, the most commonly used concentration, alters the molecular function of the hamstring graft in ACLR.

**Study Design:**

Controlled laboratory study.

**Methods:**

Surplus hamstring tendon collected after routine ACLR surgery was used for in vitro cell culture and ex vivo tissue experiments. Vancomycin was used at 5 mg/mL in RPMI or saline diluent to treat cells and tendon tissue, respectively, with diluent control conditions. Cell viability at 30, 60, and 120 minutes was assessed via colorimetric viability assay. Tendon cells treated with control and experimental conditions for 1 hour was evaluated using semiquantitative reverse transcription analysis, immunohistochemistry staining, and protein quantitation via enzyme-linked immunosorbent assay for changes in apoptotic, matrix, and inflammatory gene and protein expression.

**Results:**

Vancomycin treatment at 5 mg/mL significantly reduced tenocyte viability in vitro after 60 minutes of treatment (*P* < .05); however, this was not sustained at 120 minutes. Vancomycin-treated tendon tissue showed no significant increase in apoptotic gene expression, or apoptotic protein levels in tissue or supernatant, ex vivo. Vancomycin was associated with a reduction in inflammatory proteins from treated tendon supernatants (IL-6; *P* < .05).

**Conclusion:**

Vancomycin did not significantly alter the molecular structure of the hamstring graft. Reductions in matrix protein and inflammatory cytokine release point to a potential beneficial effect of vancomycin in generating a homeostatic environment.

**Clinical Relevance:**

Vancomycin ACL wrap does not alter the molecular structure of the ACL hamstring graft and may improve graft integrity.

Anterior cruciate ligament (ACL) reconstruction (ACLR) using patellar and hamstring tendon grafts is a relatively routine and successful surgical procedure, with low reported levels of infection (0.14%-1.8%).^9,18,31^ As with all orthopaedic surgery involving implants or grafts, it is routine to administer prophylactic antibiotics before the procedure to reduce the risk of infection and septic arthritis, potentially serious complications.^10^ Although the ideal timing of administration has been debated, it is common practice to administer them before skin incision and tourniquet inflation.^7,8^

The most common pathogen cultured in synovial fluid after ACLR infection is coagulase negative *Staphylococcal* species, generally thought to be a contamination of the graft from either patient skin or graft preparation.^23^ This suggests that targeting the graft, as a source of infection, with antimicrobials would be an efficient way to reduce postoperative infection rates.

Vancomycin is a glycopeptide antibiotic originally introduced to treat methicillin-resistant *Staphylococcus aureus* and coagulase negative *Staphylococcal* species with proven bactericidal activity. The minimum inhibition concentration required for *Staphylococcus aureus* is 0.5 μg/mL and is in the range of 0.25 μg/mL to 2.0 μg/mL for other bacteria.^3^ It is widely used in many forms in orthopaedic surgery (bone cement in routine arthroplasty, intravenous (IV) infusion to treat prosthetic joint infection, and topical powder on spinal surgery wounds), with results showing that it reduces infection significantly.^21,24^ A number of other surgical specialties utilize antibiotics and antimicrobials, either topically on wounds or in solution to immerse and wrap implants before surgery, and reports have shown significant reductions in infection.^11,30,33^ Cardiothoracic surgery has been at the forefront of this, with surgeons applying vancomycin powder to sternotomy wounds following the results of a seminal paper in 1989^33^ and now implanting cardiac electronic devices enveloped in a degradable antimicrobial sleeve.^30^

The specific practice of wrapping the donor graft in a vancomycin-soaked sterile swab before insertion in ACLR surgery has steadily increased since 2012 when the “vancomycin wrap” was first described.^34^ Since then, there have been a number of published studies and reviews that have demonstrated significantly reduced postoperative infection rates in ACLR.^5,14,19,25-27,34^

The molecular effects of vancomycin on human and animal periarticular tissues, including tendon, has a limited evidence base. Porcine chondrocyte death was significantly increased after exposure to vancomycin at doses of 5 mg/mL or higher.^29^ A human in vitro study has shown vancomycin toxicity to chondrocyte and osteoblast-like cells, via a reduction in cell DNA, at doses of 250 μg/mL and 125 μg/mL for osteoblast and chondrocytes, respectively, after 48 hours of treatment.^4^ Two further studies have concluded that vancomycin is not toxic at doses up to 16 μg/mL for 36 hours in an ex vivo chondrocyte model^12^ and up to 1000 μg/mL for 72 hours in an in vitro osteoblast model.^13^ Porcine tendon models show that vancomycin is effective at eliminating bacterial contamination at 5 mg/mL after 20 minutes of soaking and that it has no effect on the biomechanical properties at doses up to 10 mg/mL.^28^ Bovine studies have demonstrated that tendon can act as a reservoir for vancomycin, releasing the antibiotic for up to 24 hours into the joint, changing a source of infection into an intra-articular prophylactic vehicle.^15^

The current study aimed to determine whether treatment with vancomycin at 5 mg/mL, the clinically used concentration, alters the structural or molecular function of the hamstring graft in ACLR using human tendon in vitro and ex vivo models.

## Methods

### Tissue Collection and Preparation

Surplus human tendon tissue was obtained during surgery for ACL hamstring reconstruction. Patients were screened and the tissue discarded if there was a history of surgery to that joint, infection, or malignancy. The tissue was used for ex vivo and in vitro experiments as outlined below. Tissue collection complied with local research ethics committee approval. All tissue was collected under ethics REC 14/WS/1035.

### Tissue Culture

Tenocyte cells were derived from surplus hamstring tendon tissue. Cell culture was maintained for 28 days in RPMI supplemented with 10% fetal calf serum, 100 U/mL penicillin, 100 μg/mL streptomycin (Gibco), at 37°C in a humidified atmosphere of 5% CO_2_. Cells were passaged with trypsin at subconfluency and used at second or third passage for in vitro experiments.

Surplus tendon tissue from separate donors was cut into 1-cm sections for ex vivo experiments. These sections were treated with vehicle control and experimental conditions for 1 hour at 37°C in a humidified atmosphere of 5% CO_2._ Supernatants were aspirated and stored for future protein analysis and replaced with RPMI for 16 hours of culture to allow for gene expression changes and cytokine release. After the 16-hour incubation, supernatants were aspirated and stored for future analysis and the tendon tissue was transferred to RNALater (Ambion) or 4% buffered formalin for quantitative polymerase chain reaction (qPCR) and immunohistochemistry analysis, respectively.

### Cell Viability Assay

To assess the effect of vancomycin on tenocytes, cells were exposed to the clinically relevant concentration of 5 mg/mL of vancomycin for up to 120 minutes. The in vitro effect was evaluated using 3-(4,5-dimethylthiazol-2-Yl)-2,5-diphenyltetrazolium bromide cell viability assays (MTT; Sigma Aldrich). Tenocytes were plated at 25,000 cells/well in a 24-well plate for 48 hours at 37°C, 5% CO_2_, before use. The culture media was aspirated and discarded and replaced with RPMI control and experimental conditions, vancomycin 5 mg/mL in RPMI diluent for 30, 60, and 120 minutes at 37°C, 5% CO_2_. Supernatants were aspirated and replaced with sterile filtered MTT for 3 hours. Cells were then washed with sterile RPMI and incubated with DMSO for 10 minutes to dissolve the formazan product. This was transferred into a 96-well plate (100 μL per well in duplicate) and read on an MTX TC II microplate reader (Dynex Technologies) at 540 nm.

### Gene Expression Analysis via qPCR

Vancomycin-treated, vehicle control, and untreated tendon explant tissue in RNALater (Ambion) from 5 donors was used for qPCR analysis. The untreated condition allowed for any gene expression changes already present in the tendon to be evaluated. RNA was extracted using PureLink RNA Mini Kit (Thermo Fisher) as per the manufacturer’s instructions. The High Capacity cDNA Reverse Transcription Kit (Thermo Fisher) was used to prepare cDNA as per the manufacturer’s instructions on a thermal cycler (Applied Biosystems). PCR analysis was performed with PowerUP SYBER Green Master Mix (Thermo Fisher), in duplicate. Primers (Integrated DNA Technologies) are listed in Appendix 1 (available in the online version of this article). The gene expression of 9 genes involved in the intrinsic and extrinsic apoptotic pathways and extracellular matrix protein collagen 1α were assessed.

### Enzyme-Linked Immunosorbent Assay (ELISA)

Quantitation of the inflammatory proteins interleukin 6 (IL-6), interleukin 8 (IL-8), MMP3, CCL2; extracellular matrix protein collagen 1α; and mitochondrial signally apoptotic protein cytochrome C were determined in supernatants from control and treated tendon explants with vancomycin 5 mg/mL in 0.9% saline diluent, using ELISAs as per the manufacturer’s instructions (IL-6, IL8, MMP3, CCL2, Cytochrome C Invitrogen Cytoset; Thermo Fisher; and Pro Collagen 1α DuoSet; R&D Systems). Samples were run in duplicate on a 96-well plate and read on an MTX TC II microplate reader (Dynex Technologies).

### Caspase 3 Luminescence

Caspase 3 is an enzyme common to both intrinsic and extrinsic apoptosis pathways. Levels of cleaved caspase 3 in supernatants from treated and control tendon explants were determined by luminescence assay (Caspase-GLO 3/7; Promega) as per the manufacturer’s instructions. Supernatant was diluted 1:1 with Caspase-GLO 3/7 reagent, incubated for 1 hour at room temperature, and the luminescence detected on a MicroBeta TriLux 1450 plate reader (Perkin Elmer Life Sciences). All samples were run in duplicate.

### Histology and Immunohistochemistry

Treated and control tendon explant tissue, fixed in 4% buffered formalin and embedded in paraffin, was immunohistochemically stained for caspase 3 (Cell Signaling Technology). Sections of tissue were cut to 5-μm thickness (Leica Microsystems), paraffin removed by xylene, and rehydrated in graded alcohol.

Endogenous peroxidase activity was quenched with 3% (vol/vol) H_2_O_2_, and nonspecific antibody binding was blocked with 2.5% horse serum in tris-buffered saline (TBST) buffer for 30 minutes. Antigen retrieval was performed in 0.01 M citrate buffer for 25 minutes in a microwave. Tissue sections were incubated with primary antibody diluted 1:125 in 2.5% (w/v) horse serum/TBST overnight at 4°C. After 2 washes, the slides were incubated with a Vector ImmPRESS Reagent kit as per the manufacturer’s instructions for 30 minutes. The slides were washed and incubated with Vector ImmPACT DAB chromagen solution (1 drop per 1 mL diluent) for 30 seconds, followed by extensive washing with water. Finally, the sections were counterstained with hematoxylin, dehydrated in graded alcohol, and mounted in DPX with a coverslip.

Tissue sections were imaged under a light microscope (DP22; Olympus) at 10× and 40× magnification and compared with Isotype controls. Quantification of staining was performed in 2 stages. First, semiquantitative staging was performed on 5 random high-powered fields, grading the percentage of positively stained cells in the filed using the Modified Bonar Score (grade 0, no staining; grade 1, 1%-10% of cells stained positive; grade 2, 10%-20% of cells stained positive; grade 3, >20% of cells stained positive).

Second, the total number of positive and negative stained cells in each of the 5 high-powered fields was counted to generate the mean percentage positive for stained cells.

### Statistical Analysis

GraphPad Prism software (Version 6) was used for all statistical analyses, and data were reported as mean and standard error of the mean unless stated otherwise. Comparisons between treatment groups were made using 1-way analysis of variance or Student *t* test. Multiple comparisons were performed between the untreated tendon and tendon treated with vancomycin in saline compared with tendon in saline alone, as this is current clinical practice.

## Results

### Cell Viability and Apoptosis

**Figure 1.**
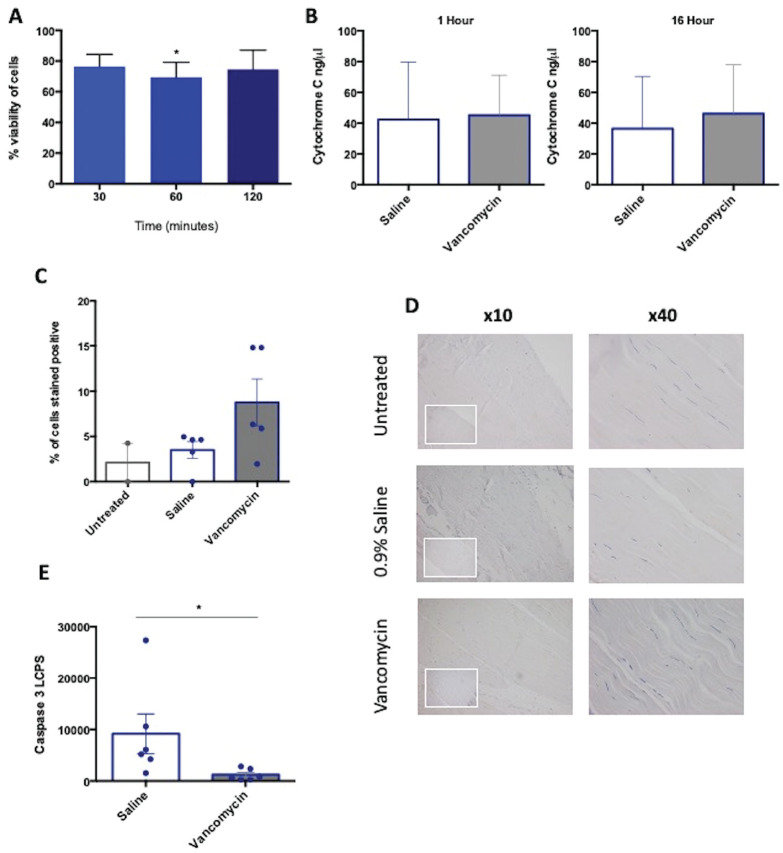
(A) Viability of tenocytes after vancomycin treatment. Data are expressed as percentage change in cell viability compared with untreated cells. N = 6, 30 and 60 minutes; N = 4, 120 minutes; analysis of variance, *P < .05. (B) Cytochrome C quantitative enzyme-linked immunosorbent assay on tendon supernatants, after 1 hour of vancomycin treatment. N = 3. (C) Quantitative expression of caspase 3, mean percentage of cells positive per treatment condition based on 5 high-power fields. N = 5. (D) Caspase 3 immunohistochemistry staining of human tendon explant, treated with experimental conditions for 1 hour, isotype rabbit IgG bottom smaller image shown in left corner of x10 image. (E) Caspase 3 luminesce in tendon supernatants after 1 hour of vancomycin treatment. N = 6; *P < .05. Data are shown as mean ± SEM.

Vancomycin treatment for 60 minutes resulted in a small but significant reduction in tenocyte viability, in vitro, compared with RPMI-treated control cells (*P* < .05). However, this was not the case with treatment at 30 and 120 minutes (Figure 1A).

Although there was only a limited effect of vancomycin on tenocyte viability, we assessed the cellular effect of vancomycin on the tendon graft by evaluating apoptotic proteins cytochrome C and caspase 3. After the ex vivo treatment, no significant increase of cytochrome C in supernatants of tendon explants was measured at 1 hour after treatment with vancomycin compared with saline (Figure 1B). In addition, there was also no significant increase in cytochrome C measured in supernatants of explant tendon at 16 hours after the 1-hour exposure to vancomycin (Figure 1B).

We assessed caspase 3 expression and protein quantitation in supernatant by ELISA and tissue by immunohistochemistry (IHC) staining, after vancomycin treatment. IHC staining showed that vancomycin treatment resulted in no disruption to the uniform collagen structure of tendon and suggested a trend toward more positive staining for caspase 3 in the vancomycin-treated tissue, but this was not significant (Figure 1, C and D). However, there was a significant reduction in caspase 3 in tendon explant supernatants after vancomycin treatment compared with saline-treated control explants (Figure 1E).

**Figure 2.**
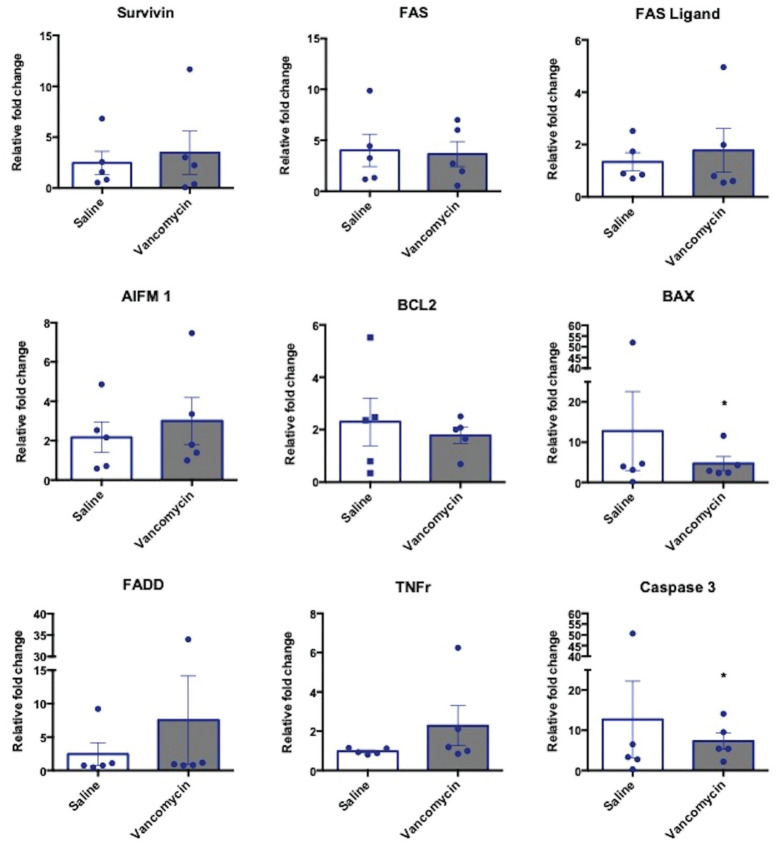
Vancomycin does not induce apoptosis in tendon anterior cruciate ligament graft. AIFM1, BAX, TNFr, Caspase 3, FADD, FAS, FAS ligand, Survivin, and BCL2 gene expression in tendon explant at 16 hours after 1-hour vancomycin treatment. Data are shown as mean ± SEM, normalized to the untreated tendon, relative to the housekeeping gene. N = 5; *P < .05 vs untreated.

The expression of 9 apoptotic genes was also measured after ex vivo treatment of tendon with experimental conditions. All results were normalized to fresh untreated graft tendon. There was no significant increase in pro-apoptotic genes with vancomycin treatment compared with untreated or saline-treated tendon (Figure 2). There was a significant reduction in expression of caspase 3 and BAX with vancomycin treatment compared with untreated explants (Figure 2).

### Matrix Protein Expression

**Figure 3.**
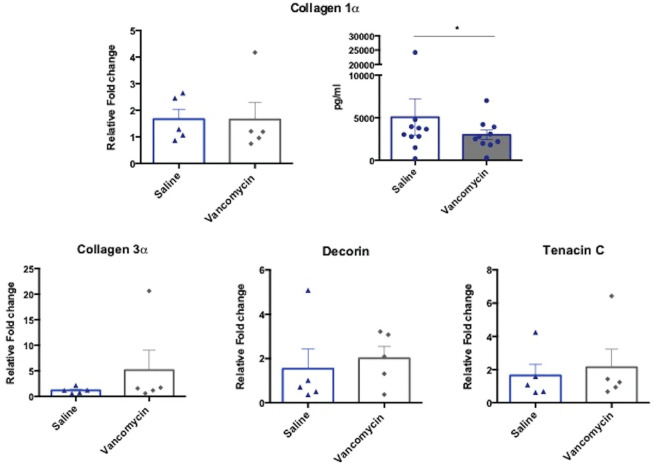
Vancomycin matrix gene and protein expression in tendon anterior cruciate ligament graft. Collagen1α, Collagen3α, Decorin, and Tenacin C gene expression in tendon explant at 16 hours after 1-hour vancomycin treatment. Data are shown as mean ± SEM, normalized to the untreated tendon, relative to the housekeeping gene. N = 5. Collagen 1α protein is measured by quantitative enzyme-linked immunosorbent assay. The tendon was treated for 1 hour, then incubated for 16 hours. Data are shown as mean ± SEM. N = 10; *P < .05.

Vancomycin treatment caused no significant changes in tendon matrix gene expression by measuring the expression of genes collagen 1α, collagen 3α, decorin, and tenascin C. There was a significant reduction in collagen 1α protein in supernatants from tendon treated with vancomycin compared with those treated with saline alone (Figure 3).

### Inflammatory Protein Release

**Figure 4.**
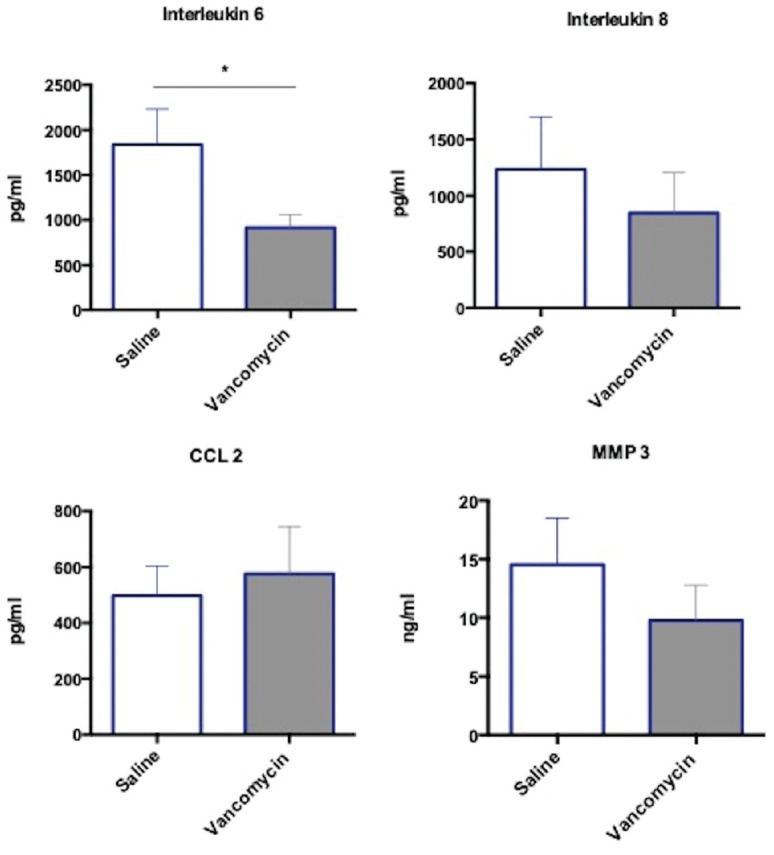
Inflammatory protein expression in tendon anterior cruciate ligament graft after vancomycin treatment. Interleukin 6 and 8, MMP3, and CCL2 protein in tendon supernatant, measured by quantitative enzyme-linked immunosorbent assay. The tendon was treated for 1 hour, then incubated for 16 hours. Data are shown as mean ± SEM. N = 10; *P < .05.

Ex vivo vancomycin treatment of tendon showed a significant reduction in the level of inflammatory cytokine IL-6 being released into the supernatant when compared with saline treatment (Figure 4). No difference was observed in CCL2, and although not statistically significant, there was a trend toward a reduction in IL-8 and MMP3 measured in supernatants of vancomycin-treated explants compared with saline.

## Discussion

The first description of the “vancomcyin wrap” for ACLR was in 2012; since then, a number of studies have demonstrated its effectiveness in combination with usual IV antibiotic prophylaxis at reducing postoperative infection rates to almost 0%.^5,14,19,25-27,34^ One study reported infection rates in the control group, treated with prophylactic IV antibiotics alone, as 1.4% (n = 285) compared with 0% in patients who were treated with prophylactic IV antibiotics in combination with vancomycin wrap (n = 870).^34^ A further study that conducted a postoperative follow-up of 1300 consecutive ACLRs using the vancomycin wrap in combination with standard IV antibiotic prophylaxis reported zero postoperative infections against a retrospective control group (n = 240) that reported infections in 2.4% of patients.^27^ A more recent study that looked at 1640 patients found the use of a vancomycin wrap during ACLR was associated with a 10-fold reduction in postoperative infection (0.1% vs 1.2%).^5^

Our study is the first to evaluate the molecular and structural effects that vancomycin has on a human hamstring graft for ACLR. The results of the current work indicate that vancomycin appears safe and has no detrimental effects on the cellular or molecular structure of the tendon graft used for ACLR.

The addition of vancomycin at the commonly used clinical dose did result in a reduction in tenocyte viability at 60 minutes. It is common in an in vitro model to experience biological variability and we did not see this reduction sustained after 30 or 120 minutes of treatment. Previously published literature that used the same assay to assess chondrocyte viability found vancomycin to be safe at doses up to 16 mg/L over 36 hours of treatment,^12^ above the minimum inhibitory concentration for *Staphylococcus aureus*.^3^ It remains unclear from the published literature how much of the antibiotic is absorbed both by the sterile swab and by the tendon itself and therefore what concentration the tenocytes are exposed to. One study reported that the sterile swab absorbs an average of 7 mL of the 100-mL solution containing vancomycin at 5 mg/mL.^34^ Bovine studies specifically looking at absorbance and elution of vancomycin from tendon have demonstrated that both of these characteristics are affected by graft size and rinsing,^15^ which occurs in clinical practice once implanted, with arthroscopic washout of the joint. It is challenging to develop a model that accounts for all of these variables. We therefore submerged uniform size tendon explant pieces in vancomycin or saline for 1 hour. This eliminated any effect drying may have had on the tendon and was long enough to encompass reported average times from graft harvest to implantation of 30 minutes (range, 28-43 minutes).^16^ The graft was then cultured for up to 16 hours after 1 hour of treatment to assess the effect of vancomycin on tendon gene expression and cytokine release. We used this as our “surgical scenario” model and believe this accurately reflects current practice and gives confidence that our molecular findings translate to everyday clinical practice.

Treatment with vancomycin did not appear to initiate cell death as there was no increase in cytochrome C in supernatant and no increase in caspase 3 protein in explant tissue. Interestingly, there may be a small but beneficial effect of vancomycin in decreasing caspase 3 in supernatants from treated tendon, mirroring the significant reduction in the gene expression of caspase 3. Furthermore, there was little evidence that vancomycin induced any upregulation in apoptotic gene expression compared with saline-treated grafts.

The primary function of the tendon graft in ALCR is to transfer mechanical load. Although the current study did not directly measure the biomechanical properties of the tendon graft after vancomycin treatment, histological studies indicate vancomycin treatment does not affect the integrity of the graft structure. Furthermore, exploration of the effect of vancomycin treatment on the matrix component of the graft after treatment demonstrated no significant difference in matrix gene expression compared with untreated tendon or saline-treated tendon. It is important to note, however, that there was a small but significant reduction in collagen 1α protein release in vancomycin-treated tendon supernatants compared with saline treatment. It is postulated that this may demonstrate lower collagen 1α protein breakdown from the tissue explant into the supernatant after vancomycin treatment and thus may reflect a positive effect of vancomycin treatment.

Vancomycin, as well as being antimicrobial, has also been shown to have immune modulating potential. In inflammatory mediated conditions, it has been shown to act via alterations in the host T-cell population and lead to reduced inflammation.^1^ In a sepsis model, however, vancomycin was found to have proinflammatory effects on the innate immune system.^6^ We therefore investigated the inflammatory effect vancomycin had on tendon. Our results show that vancomycin treatment reduced inflammatory cytokine release from tendon compared with saline alone, significantly IL-6. Previous work has shown that inflammation has a negative effect on the health of a tendon and IL-6 is involved in the development of tendinopathy.^22^ The “vancomycin wrap” has the potential to create an anti-inflammatory environment.

These results suggest vancomycin treatment is not detrimental to the cellular viability of the tendon graft but may indeed have small beneficial effects pointing toward a homeostatic molecular environment to encourage graft viability. The reduction in collagen 1α release and inflammatory cytokine release indicate reduced cellular stress and may be linked with the reduction seen in the apoptotic gene expression of caspase 3. A recent retrospective cohort study reviewed 1779 patients who underwent ACLR with vancomycin soaking of the hamstring graft employed in 853 of these cases. Over a 5-year period, whereby 100 patients were randomly selected for follow-up each year, graft failure and re-rupture rates were reported to be significantly lower in the vancomycin wrap group; 8 failures out of 257 versus 16 failures out of 167 patients in the control group (*P* < .01).^25^

Risk factors and prevention strategies for postoperative infection in ACLR are multifactorial, with longer surgical times and higher body mass index both shown to be independent predictors.^5^ When infection rates are already low, 1 intervention alone may not consistently alter outcomes. This was the conclusion in 1 study assessing the efficacy of local vancomycin to spinal surgery wounds that reported no difference in infection rates versus usual IV antibiotic prophylaxis (1.61% vs 1.68%).^32^ Furthermore, infection after ACLR can be caused by multiple pathogens that may not be susceptible to vancomycin and the increasing use of 1 antibiotic as local prophylaxis may begin to select out for non-*Staphylococcal* infections, which does not constitute resistance but may have implications for clinical treatment protocols should infection occur.^17^

Antibiotic resistance is an important consideration as an increasing number of antibiotics are being used in the perioperative period as prophylaxis. Data from cardiothoracic surgery using vancomycin paste in sternotomy wounds have not found antibiotic resistance to be an issue given that topical use does not lead to therapeutic serum levels of the drug.^20^ Animal studies have demonstrated that once wrapped in a vancomycin solution, the ACL graft can act as a reservoir, continuing to release the drug over 24 hours at gradually decreasing levels, below a dose considered toxic to cells.^15^ Exposing tissues to prolonged subtherapeutic levels of a drug is a known way to establish resistance.^2^ Future evaluation on the safety of the vancomycin wrap in ACLR could include serum and synovial drugs levels after surgery to investigate this.

There are limitations to this work, primarily with the tendon explant model. There are many variables involved in the vancomycin wrap during surgery: the volume of antibiotic absorbed by the swab and by the graft, both influenced by the volume of tissue and rinsing during the arthroscopic procedure, and the effect of drying of the graft before implantation under the lamina flow. It is challenging to create a model that encompasses all of these variables. We believe the model we used accurately reflects the effect of the antibiotic alone on the graft. Further studies and clinical follow-up are recommended to investigate the tendon graft biomechanical properties after treatment, if there is any association with reduced graft failure and any indications of antibiotic resistance.

## Conclusion

No detrimental effect of vancomycin treatment was found in terms of cellular apoptosis, matrix protein expression, and inflammatory cytokine levels, compared with saline treatment, in hamstring ACL graft. Additionally, data from the current study provide minor indications for the beneficial effect of vancomycin treatment on ACL graft integrity by promoting a homeostatic molecular environment.

## Supplemental Material

sj-pdf-1-ajs-10.1177_0363546520981570 – Supplemental material for Vancomycin Wrap for Anterior Cruciate Ligament Surgery Click here for additional data file. Supplemental material, sj-pdf-1-ajs-10.1177_0363546520981570 for Vancomycin Wrap for Anterior Cruciate Ligament Surgery by Caroline M. Atherton, Simon J. Spencer, Katy McCall, Emma Garcia-Melchor, William J. Leach, Michael Mullen, Brian P. Rooney, Colin Walker, Iain B. McInnes, Neal L. Millar and Moeed Akbar in The American Journal of Sports Medicine
