# Supplementary material for: Vancomycin Wrap for Anterior Cruciate Ligament Surgery: Molecular Insights
Source: Am J Sports Med. 2021 Jan 6;49(2):426–34. doi: 10.1177/0363546520981570 (PMC7859666; doi:10.1177/0363546520981570)
Supplement: sj-pdf-1-ajs-10.1177_0363546520981570 – Supplemental material for Vancomycin Wrap for Anterior Cruciate Ligament Surgery [file sj-pdf-1-ajs-10.1177_0363546520981570.pdf]

Supplementary Appendix A: qPCR Primers

Primer	Forward (5' – 3')	Reverse (5' – 3')
**GAPDH**	TCG ACA GTC AGC CGC ATC TTC TTT	ACC AAA TCC GTT GAC TCC GAC CTT
**Collagen 1α**	CAA TGC TGC CCT TTC TGC TCC TTT	CAC TTG GGT GTT TGA GCA TTG CCT
**Collagen 3α**	TAT CGA ACA CGC AAG GCT GTG AGA	GGC CAA CGT CCA CAC CAA ATT CTT
**Decorin**	CGC CTC ATC TGA GGG AGC TT	TAC TGG ACC GGG TTG CTG AA
**Tenascin C**	GTG CAA GGA CGT ACC AC	CTT TGG CTG GGT TGC TTG AC
**AIFM1**	CAG TCA ATG TTC TGG AGT GAT TTG	TTG TCT TGT GCA GTT GCT TT
**BAX**	GGA GCT GCA GAG GAT GAT TG	AGT TGA AGT TGC CGT CAG AA
**BCL2**	AGG CTG GGA TGC CTT TGT	GAC TTC ACT TGT GGC CCA GAT A
**Caspase 3**	TCA TTA TTC AGG CCT GCC GTG	TGG ATG AAC CAG GAG CCA TCC
**FADD**	GGA AGA AGA CCT GTG TGC AG	GCA TCT TGG TGT CTG AGA CTT T
**FAS**	AGG GAT TGG AAT TGA GGA AGA	CTA GCT TTC CTT TCA CCT GGA
**FAS Ligand**	ATA GGC AAG TCC AAC TCA AGG	CAC AAG GCC ACC CTT CTT AT
**Survivin**	GAA CTG GCC CTT CTT GGA G	CTT GAA GCA GAA GAA ACA GCT
**TNFr**	GAA GAA CCA GTA CCG GCA TTA T	TGC ACA CGG TGT TCT GTT
